# Endovascular Treatment of Dural Arteriovenous Fistulas of the Anterior or Posterior Condylar Vein: A Cadaveric and Clinical Study and Literature Review

**DOI:** 10.1007/s00062-018-0669-1

**Published:** 2018-02-05

**Authors:** V. Hellstern, M. Aguilar-Pérez, S. Schob, P. Bhogal, M. AlMatter, P. Kurucz, A. Grimm, H. Henkes

**Affiliations:** 1 Neuroradiological Clinic, Neurocenter, Klinikum Stuttgart, Stuttgart, Germany; 2 Department of Diagnostic and Interventional Radiology, University Hospital of Leipzig, Leipzig, Germany; 3 Department of Neurosurgery, Neurocenter, Klinikum Stuttgart, Stuttgart, Germany; 4 Department of Otorhinolaryngology, Head and Neck Surgery, Semmelweis University, Budapest, Hungary; 5 Laboratory for Applied and Clinical Anatomy, Department of Anatomy, Histology and Embryology, Semmelweis University, Budapest, Hungary; 6 Medical Faculity, University Duisburg-Essen, Essen, Germany

**Keywords:** Dural arteriovenous fistula, Condylar vein, Endovascular, Transvenous embolization, Coils

## Abstract

Dural arteriovenous fistulas (DAVF) involving the anterior and posterior condylar vein at the skull base are rare but important to recognize. Due to the highly variable anatomy of the venous system of the skull base, detailed anatomical knowledge is essential for correct diagnosis and appropriate treatment of these lesions. In this report we review the normal anatomy of the condylar veins and describe rare and, to our knowledge, not previously reported anatomical variants. We also highlight the treatment modalities for these lesions with focus on the endovascular transvenous occlusion based on four consecutive cases from our center.

## Introduction

Dural arteriovenous fistulas (DAVF) are acquired pathological arteriovenous connections involving vessels that usually supply the meninges [1, 2]. They are most commonly found in the posterior fossa and usually involve the transverse and sigmoid sinus [1, 3, 4]. DAVFs of the anterior and posterior condylar veins are rare [3, 5], but important to recognize as these entities can have significant clinical implications. Correct diagnosis of these lesions can, however, be challenging due to the diverse anatomical variants of the venous system at the skull base. Choosing the best treatment strategy thus requires extensive understanding of the complex vascular architecture of DAVFs in the suboccipital region.

The anatomy of the venous system of the skull base has been described in several studies [5–10], but rare and not previously reported variants can be encountered in the clinical practice due to the extremely variable anatomy of this region. In this report we describe the normal anatomy of the condylar veins as well as anatomical variations which could be important to recognize. We also present the clinical and radiological findings of four cases of DAVFs involving the anterior condylar vein and review the relevant literature.

## Methods of the Anatomical Study

In this study 20 human cadaveric specimens (40 suboccipital regions) were examined. The cadavers were donated for research and medical education to the Department of Anatomy, Histology and Embryology, Semmelweis University, Budapest, Hungary.

The angioarchitecture and bony relations in and around the occipital condyles were examined on macerated anatomical specimens. In 10 out of 20 cases non-fixed, fresh cadaveric heads were prepared as follows: the external and internal carotid arteries as well as the internal jugular veins and the sigmoid sinuses were selectively cannulated. The arteries were filled with red and the veins with blue colored Akemi Akepox® (Akemi, Germany) adhesive mixed with Duracryl® Plus (SpofaDental, Czech Republic) self-curing base resin, respectively. This was followed by anatomical dissections of the suboccipital region through far-lateral directed surgical approaches. As the last step of preparation the soft-tissues were completely macerated at a temperature between 35–50 °C and the bony and vascular relations were examined and photodocumented.

The soft tissue relations of the region were studied on 10/20 cadaveric heads fixed in 4% buffered formaldehyde solution. The occipital condyles were removed on both sides as blocks including the entire examined area. After dissection of the soft-tissues photodocumentation was performed.

## Results of the Anatomical Study

### Anatomy of the Occipital Condylar Region

**Fig. 1:**
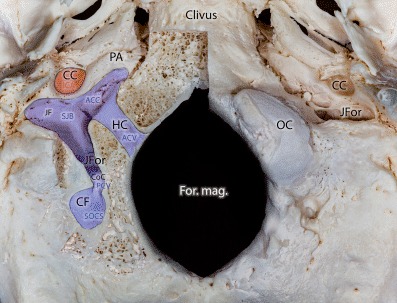
Bony relations of the occipital condylar region on the external cranial base. On the right side of the specimen the occipital condyle was partly removed to show the entire length of the hypoglossal and condylar canals as well as their junction around the jugular fossa. *ACC* anterior condylar confluence, *ACV* anterior condylar vein, *CC* carotid canal, *CF* condylar fossa, *CoC* condylar canal, *For. mag.* foramen magnum, *HC* hypoglossal canal, *ICA* internal carotid artery, *JF* jugular fossa, *JFor* jugular foramen, *OC* occipital condyle, *PA* petrous apex, *PCV* posterior condylar vein, *SJB* superior bulb of the internal jugular vein, *SOCS* suboccipital cavernous sinus

The venous portion of the jugular foramen is separated from the sigmoid sinus sulcus by a thin bony ridge called the terminal margin. Ventral to this structure the jugular fossa is located, which is filled by the superior bulb of the internal jugular vein (SJB). The condylar canal (CoC), a small bony canal that passes through the occipital condyle dorsoventrally along its longer axis, terminates cranially or caudally of the terminal margin (Fig. 1).

**Fig. 2:**
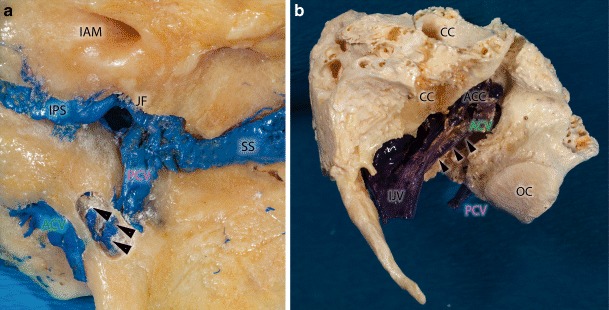
Macerated bony specimens presenting the lateral suboccipital region with preserved veins (filled with *blue* color). **a** Medial surface of the petrous bone on the right side. In this special case we could observe a venous connection between the anterior and posterior condylar vein. The bony canal of this bridging vein was drilled from medial (*arrowheads*). *ACV* anterior condylar vein, *IAM* internal auditory meatus, *IPS* inferior petrosal sinus, *JF* jugular foramen, *PCV* posterior condylar vein, *SS* sigmoid sinus

The posterior condylar vein (PCV), which is an emissary vein connecting the extracranial and intracranial venous system (Figs. 1 and 2a, b), runs through the CoC. Extracranially the PCV originates from the suboccipital cavernous sinus (SOCS), which is located within the condylar fossa. Intracranially the PCV drains into the SJB.

**Fig. 3:**
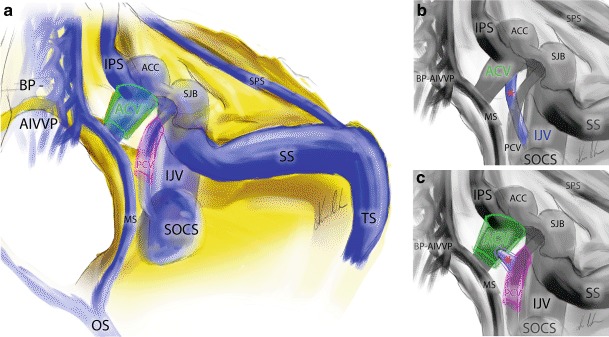
Schematic overview of the dural sinuses and veins in the suboccipital and petrous region on the right side. **a** Relations and connections of the anterior condylar (*green*) and posterior condylar (*purple*) veins. **b** Direct venous drainage between the anterior condylar and internal jugular veins (*red star*). **c** A small sinus (*red star*) within its own bony canal (*blue line*) connect the anterior condylar (*green*) and posterior condylar (*purple*) veins. *ACC* anterior condylar confluence, *ACV* anterior condylar vein, *BP-AIVVP* basilar plexus-anterior internal vertebral venous plexus, *IJV* internal jugular vein, *IPS* inferior petrosal sinus, *MS* marginal sinus, *OS* occipital sinus, *SJB* superior bulb of the internal jugular vein, *SOCS* suboccipital cavernous sinus, *SPS* superior petrosal sinus, *SS* sigmoid sinus, *TS* transversal sinus

The anterior condylar vein (ACV) is located within the hypoglossal canal (HC) and passes through the occipital condyle mediolaterally, perpendicular to the longer axis of the condyle (Fig. 1). The intracranial end of the ACV is connected to the area of the anterior internal vertebral venous plexus (AIVVP), basilar plexus and the marginal sinus (Fig. 3a). The extracranial ending of the ACV terminates in the anterior condylar confluence (ACC). The ACC is a venous pouch located at the extracranial opening of the hypoglossal canal and drains directly into the SJB. The HC and the CC as well as the ACV and PCV within them are situated nearly perpendicularly to each other (Fig. 3a).

In two of the 40 examined cadaveric samples we could verify a bony communication between the HC and CoC within the occipital condyle with a small venous anastomosis connecting the intracondylar portions of the ACV and PCV (Figs. 2a and 3c). In two other cases the ACV drained directly into the internal jugular vein bypassing the ACC and the SJB (Figs. 2b and 3b).

**Fig. 4:**
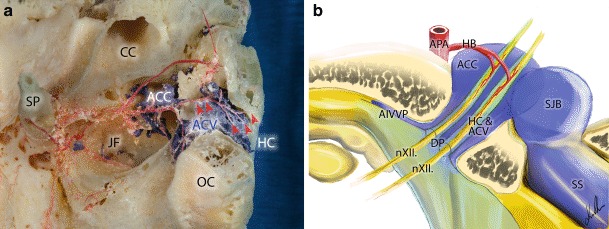
Anatomical relations within and around the hypoglossal canal on the right side. **a** Macerated bony specimen of the hypoglossal canal. Note the hypoglossal branches of the ascending pharyngeal artery within the hypoglossal canal (*red arrowheads*). **b** The two presented trunks of the hypoglossal nerve enter the hypoglossal canal through separate dural pores. The trunks have their own separate dural sheaths within the canal surrounded by a dural venous sinus called the anterior condylar vein. Each trunk has its own arterial blood supply through the hypoglossal artery originating from the neuromeningeal branch of the ascending pharyngeal artery. The anterior condylar vein drains into the anterior condylar confluence located at the extracranial opening of the hypoglossal canal. This venous pouch is connected to the superior bulb of the internal jugular vein. *ACC* anterior condylar confluence, *ACV* anterior condylar vein, *AIVVP* toward the anterior internal vertebral venous plexus, *APA* anterior pharyngeal artery, *CC* carotid canal, *DP* dural pores, *HB* hypoglossal branch, *HC* hypoglossal canal, *JF* jugular fossa, *nXII* hypoglossal nerve, *OC* occipital condyle, *SJB* superior bulb of the internal jugular vein, *SP* styloid process, *SS* sigmoid sinus

The arterial supply of this region is provided by the ascending pharyngeal artery (APA). The neuromeningeal branch of the APA runs parallel to the internal jugular vein towards the jugular foramen, where it bifurcates. One of the branches enters the jugular foramen and supplies the dura of the posterior fossa. The other branches enter the HC and supply the neuromeningeal structures located within the canal. The hypoglossal nerve can sometimes enter the HC via two or more dural pores. In such cases the individual trunks have their own dural sheaths along the entire length of the canal. The arterial blood supply of these individual trunks is provided by their own branches from the neuromeningeal trunk of the APA through the hypoglossal canal (Fig. 4a, b). This variation was observed in nearly half of the cadaveric samples (17 out of 40).

## Case Descriptions

Between February 2015 and March 2017 a total of 4 cases of DAVFs involving the anterior condylar vein were treated by endovascular transvenous coil embolization at our center. In one case additional transarterial embolization was performed (Table 1).

**Table 1:** Four patients with a DAVF involving the ACV, diagnosed and treated by the authors

Case	Symptoms	Classification	Feeding artery	Draining vein	Treatment/Result
#1 (51 years, M)	Tinnitus	Cognard 2a, Borden 1	Ascending pharyngeal artery both sides	Antegrade to the jugular vein + AIVVP	TVE/cured
			Posterior meningeal branches of the right vertebral artery	Retrograde to the sigmoid sinus, inferior petrous sinus, cavernous sinus, ophthalmic vein	
			Right internal maxillary artery		
			Right occipital artery		
			Meningohypophyseal and inferolateral trunk of the right internal carotid artery		
#2 (64 years, F)	Tinnitus, miosis, ptosis	Cognard 2a, Borden 1	Ascending pharyngeal artery of both sides	Antegrade to the jugular vein	TVE/cured
			Posterior meningeal branch of the left vertebral artery	Retrograde to inferior petrous sinus, cavernous sinus	
			Inferolateral trunk of the left internal carotid artery		
#3 (71 years, M) (Fig. 5)	Tinnitus	Cognard 2a, Borden 1	Ascending pharyngeal artery of both sides	Antegrade to the right jugular vein	TAE/failed TVE/cured
			Right occipital artery	Antegrade to the posterior condylar vein	
			Right internal maxillary artery	Retrograde to the right inferior petrous sinus and cavernous sinus	
#4 (79 years, M) (Fig. 6)	Tinnitus, double images, conjunctival hyperemia, protrusion of the bulb	Cognard 3, Borden 2	Left ascending pharyngeal artery	Antegrade to the left jugular vein	TVE/cured
				Retrograde to the left inferior, petrous sinus, cavernous sinus superior ophthalmic and cortical veins	

*TVE* transvenous embolization, *TAE* transarterial embolization, *AIVVP* anterior internal vertebral venous plexus

### Illustrative Cases

#### Case #3

**Fig. 5:**
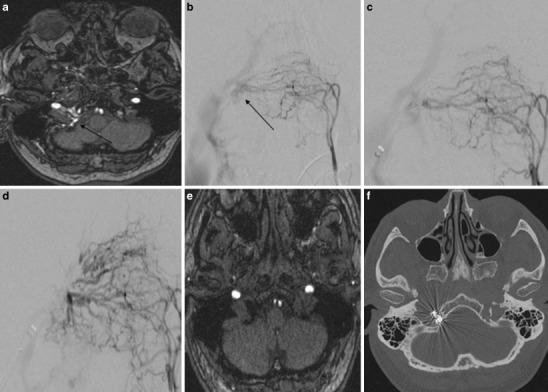
Case #3: **a** MR angiography source image from three-dimensional time-of-flight shows abnormal vascularization in the right hypoglossal canal compatible with a DAVF of the anterior condylar vein. **b** Anteroposterior (AP) view after injection of the left ascending pharyngeal artery shows a DAVF of the anterior condylar vein with a venous pouch located medially to the jugular bulb. **c** AP view after injection of the left ascending pharyngeal artery shows the microcatheter in the venous pouch and partial coil occlusion of the DAVF due to a transvenous approach. **d** AP view shows the complete occlusion of the DAVF; **e** MR angiography source image from three-dimensional time-of-flight shows no abnormal vascularization in the right hypoglossal canal anymore. **f** Axial computed tomography (CT) scan of the skull base shows the location of the coils in the right hypoglossal canal

The 71-year-old male patient presented due to right-sided pulse-synchronous tinnitus, especially at night. The cranial magnetic resonance imaging (MRI) showed increased vascular signal in the right HC suspicious for a DAVF (Fig. 5a). The catheter-based angiography confirmed the finding of a DAVF of the anterior condylar vein with arterial feeders arising from the right APA, the right occipital artery, the right internal maxillary artery and also from the left APA (Fig. 5b). The venous drainage was to the anterior condylar vein into the right jugular vein and the posterior condylar vein anterogradely with reflux into the right inferior petrous sinus and cavernous sinus (Cognard 2a, Borden 1).

Due to the reduced quality of life caused by the pulse-synchronous tinnitus the patient decided to undergo endovascular treatment.

Due to the high volume of the shunt we initially decided to perform the first treatment session transarterially over a 6Fr guide catheter that was placed in the ipsilateral external carotid artery. Multiple arterial feeders were then consecutively catheterized and occluded using N-Butyl Cyanoacrylate (NBCA) with marked reduction of the shunt. Due to the persistence of the debilitating tinnitus we decided to perform a transvenous embolization in a second treatment session.

The transvenous intervention was carried out through a transfemoral approach. A 6Fr guide catheter was tracked into the right internal jugular vein. A microcatheter was advanced to the point of the fistula at the ACV and 11 detachable coils were deployed in the ACV und ACC (Fig. 5c, d).

The post-interventional MRI scan confirmed the complete occlusion of the DAVF (Fig. 5e). A computed tomography (CT) scan of the skull base confirmed the location of the coils in the HC (Fig. 5f). The tinnitus disappeared immediately after the second treatment and the patient was discharged 3 days after the treatment.

The follow-up angiography 2 years after the treatment confirmed the persistent complete occlusion of the DAVF. The patient has also remained asymptomatic during the 2-year follow-up.

#### Case #4

**Fig. 6:**
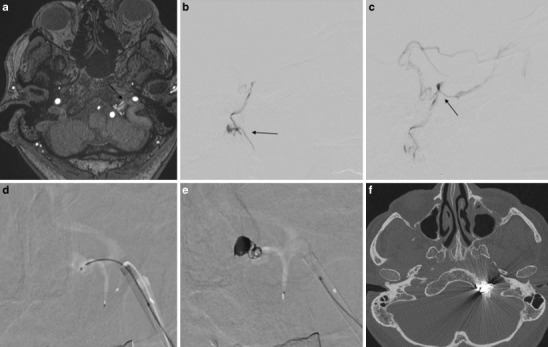
Case #4: **a** MR angiography source image from three-dimensional time-of-flight shows abnormal vascularization in the left hypoglossal canal compatible with a DAVF of the anterior condylar vein (*arrow*). **b** Lateral view after injection of the right ascending pharyngeal artery shows a DAVF of the anterior condylar vein with a venous pouch (*arrow*) located medial to the jugular bulb. **c** Lateral view after injection of the right ascending pharyngeal artery shows a DAVF of the anterior condylar vein with a venous pouch at the point of the fistula (*arrow*) and also reflux to a petrosal and sphenopalatinal cortical vein. **d** AP view shows the microcatheter in the venous pouch of the DAVF of the anterior condylar vein through a transvenous approach via the left jugular vein. **e** AP view after injection of the left ascending pharyngeal artery after coil occlusion of the left anterior condylar vein shows the complete occlusion of the DAVF. **f** Axial CT scan of the skull base confirmed the location of the coils in the hypoglossal canal

## Results of the Clinical Cases

In all cases, arterial feeders originated from the APA, mainly from both sides. Further observed arterial feeders arose from the occipital artery, dural branches of the vertebral artery and the internal carotid artery. The venous drainage patterns were also quite similar in all the cases. The venous drainage patterns were anterograde to the jugular vein and extracranial vertebral venous plexus, but also included retrograde perfusion of the inferior petrous sinus to the cavernous sinus in all of the 4 cases.

## Discussion

### Anatomy

The HC is an intraosseous canal on both sides of the foramen magnum cranially to the occipital condyle. It contains the hypoglossal nerve, the neuromeningeal branch of the ascending pharyngeal artery and the anterior condylar vein [3, 5, 10]. The ACV is a venous plexus that surrounds the hypoglossal nerve whilst it traverses the HC [5, 10]. It links the ACC to the anterior internal venous vertebral plexus (AIVVP) (Figs. 1 and 3a). Furthermore, connections to the basilar plexus are infrequently observed. In angiograms, the ACV, and hence DAVFs of the ACV and the ACC are found immediately medially, inferiorly and anteriorly to the jugular bulb (Figs. 3c and 5b).

The CoC is an intraosseous canal that connects the dorsal aspect of the jugular foramen with the condylar fossa and terminates directly dorsal to the occipital condyle [11]. Within the CoC run the PCV and meningeal branches of the occipital artery. The PCV originates from the bulb of the jugular vein, the most medial point of the sigmoid sinus or the ACC (Fig. 4b; [10, 12, 13]). It terminates in the vertebral artery venous plexus (VAVP) or deep cervical veins (DCV). As one of the largest emissary veins the PCV is an important anastomosis between the intracranial and extracranial venous systems. In angiograms, the posterior condylar vein and hence also fistulas of the PCV can be found dorsally and posteriorly to the bulb of the internal jugular vein (Fig. 4a; [10]).

### Anatomical Variants

Ota et al. [9] reported a rare variant of a PCV originating directly from the intracondylar portion of the ACV (7.4–7.7% of cases). Matsushima et al. reported the same variant in 0–3% of cases in their series [6]. In addition to this variant we found two cases where the PCV is not a branch of the ACV but where a small anastomotic vein connects the ACV and PCV through its own bony canal. In these cases the venous flow is secured though several channels. To the best of our knowledge, this variation has never been previously described. The ACV drains normally into the internal jugular vein over the extracranially located ACC [5, 14–16]. In the studied specimens we could verify the existence of a single venous branch originating from the extracranial portion of the ACV connecting directly to the internal jugular vein. This anatomical variation has also not been previously reported.

The arterial supply of the examined region is well-described in literature [17]; however, we found an interesting, previously undescribed anatomical variation of the arteries within the HC: in some cases the hypoglossal nerve is divided by dural sheaths to numerous trunks within the HC and these trunks have each their own arterial supply from the APA (Fig. 4a, b). Although there are reported cases of IX–XII cranial nerve dysfunctions after transarterial embolization of DAVFs in the region of the ACV [18], this anatomical fact is a possible explanation why hypoglossal nerve injury is not a frequently observed therapeutic injury.

### DAVFs of the Anterior and Posterior Condylar Vein

The DAVFs involving the condylar veins are rare entities. To date approximately 120 cases of DAVFs of the anterior condylar vein [5] and, to our knowledge, only 3 cases of DAVF involving the posterior condylar vein have been reported [19–21].

### Etiology

The pathophysiology of DAVFs still remains unknown. There is an increased incidence of DAVFs in patients who previously suffered a sinus thrombosis, especially in patients with a lateral DAVF [1, 2, 22]. Head trauma, prior head surgery, infection or inflammation have been discussed in the literature as other possible causes of DAVFs [1, 2]. An association between DAVFs of the ACV or PCV and sinus thrombosis, however, has not been reported in literature, to the best of our knowledge. There was no clear evidence of sinus thrombosis in any of our cases. This might suggest that condylar DAVFs may be unrelated to a previous sinus thrombosis. A more likely cause for condylar DAVFs might be due to infections or after surgery of the skull base; however further studies might be necessary to determine the underlying etiology of condylar DAVFs.

### Clinical and Radiological Findings

Typical clinical presentations of DAVFs of the ACV are pulse-synchronous tinnitus [3, 5] and ophthalmological symptoms such as chemosis, proptosis or diplopia due to venous reflux into the inferior petrosal sinus, the cavernous sinus and the superior ophthalmic vein [5, 23, 24], hypoglossal nerve palsies [5] and, in the case of cortical venous drainage, intracranial bleeding.

The arterial blood supply of the DAVFs of the ACV is provided typically by neuromeningeal branches of the APA [5] or in some cases secondary through other arteries such as the internal carotid artery, internal maxillary artery, occipital artery, medial meningeal artery or the vertebral artery [5, 15]. If arterial feeding from the APA is present, this finding makes it highly suspicious of a condylar DAVF. This is easy to detect on angiographic images and in case of doubt selective injections of the APA can be performed.

In patients with DAVFs of the anterior condylar vein it can be difficult to distinguish the exact location of the fistula due to the complex anatomy of the arterial and venous system at the skull base and the possible wide arterial supply of the fistulas [3, 5, 13]. In our experience the injection of the contralateral external carotid artery can be very helpful to get a better understanding and overview of the anatomy and the exact location of the point of the fistula. This was previously also reported by Ernst et al. [3].

The DAVFs of the PCV are a much less frequent entity and there are only 3 case reports in the literature (Table 2; [19–21]). In two of the three reported cases patients presented with pulse-synchronous tinnitus only [19, 21]. The third case presented with an acute subarachnoid hemorrhage [20]. In all three cases, arterial feeders originated from the neuromeningeal branch of the APA and the vertebral artery. In two of the three cases, additional arterial feeders were observed from the occipital artery. The venous drainage in two cases occurred through the PCV to the sigmoid sinus and paravertebral veins.

**Table 2:** The three patients with a DAVF involving the PCV, derived from the literature

Case	Symptoms	Feeding artery	Draining vein	Treatment/Result
1 (age unknown, F)	Tinnitus	Ascending pharyngeal artery	Suboccipital cavernous venous plexus	TVE/cured
		Vertebral artery	Sigmoid sinus and internal jugular vein	
		External carotid artery		
2 (54 years, M)	Tinnitus	Ascending pharyngeal artery	Sigmoid sinus	TVE/cured
		Vertebral artery bilaterally	Paravertebral veins	
		Occipital artery		
		Clival artery		
3 (43 years, M)	SAH with intraventricular hemorrhage	Ascending pharyngeal artery	Single medullary bridging vein	TAE/cured
		Vertebral artery bilaterally		

*TVE* transvenous embolization, *TAE* transarterial embolization, *SAH* subarachnoid hemorrhage

### Differential Diagnosis

An important differential diagnosis for lesions with increased vascularization around the jugular foramen are glomus tumors [25]. The main arterial feeder of these paragangliomas is also the APA [26, 27]. The differentiation between the glomus tumors and condylar DAVFs can be difficult in some cases [25]. Paragangliomas show the pathognomonic “salt and pepper”-pattern on MRI [26]. The typical angiographic finding is a relatively well defined tumor blush of the paraganglioma in contrast to condylar DAVFs [27]; however, it is important to include paragangliomas in the differential diagnosis.

### Therapeutic Options

The therapeutic options for DAVFs in general are endovascular treatment, microsurgical resection or stereotactic radiosurgery [2]. Microsurgical treatment of DAVFs has been reported to be safe and effective [2]. The success and risk of surgery depend on the location and the morphological characteristics of the DAVF; however, DAVFs at the skull base have a higher operative risk profile compared to other DAVFs. This is due to the more difficult surgical access course because of the intraosseous location of the arteriovenous shunt, the closeness to important structures such as the cranial nerves and the need to remove the condylar bone leading to possible craniocervical instability [3, 8, 28, 29].

The results of stereotactic radiosurgery of DAVFs involving the skull base were discouraging [1, 30]. Furthermore, this treatment modality carries the risk of radiation-induced damage to the brainstem and the cranial nerves and may therefore not be suitable for the treatment of condylar DAVFs [31]. The relatively long time between radiosurgery and the treatment effect is another disadvantage of radiosurgery, especially in high-grade DAVFs with extensive cortical drainage [31]. Endovascular therapy of DAVFs has become the most widely used treatment modality as it is considered to be a safe and effective method [2, 32]. It can be performed as transvenous embolization, transarterial embolization or as a combination thereof but both have the same aim of occluding the point of the fistula [2, 32].

All four presented cases were treated endovascularly. Of the four patients, three had a complete occlusion of the arteriovenous shunt after one treatment session performed as transvenous coil occlusion. One patient had to undergo a second treatment session to achieve complete occlusion of the fistula as transarterial embolization failed to occlude the DAVF. All patients reported an immediate and complete regression of the pulse-synchronous tinnitus after the occlusion of the arteriovenous shunt.

The three reported cases of DAVFs involving the PCV were treated via an endovascular approach. In one case a transarterial embolization was performed due to the extreme tortuosity of the bridging vein with an associated aneurysm. The other two patients were treated by selective transvenous coil embolization of the DAVFs. In all cases the fistula could be excluded completely [19–21].

The arterial feeders of DAVFs of the ACV and PCV mainly originate from the APA, the occipital artery and from meningeal branches of the vertebral artery [5]. These arteries have well known, dangerous anastomoses with the brainstem and therefore a primary transarterial embolization may not be suitable [17, 33]. Furthermore, the arterial branches supplying these fistulas can be small and extremely tortuous meaning that a stable distal catheter position may not be possible. For these reasons our preferred approach is transvenous embolization with coils. As these venous pathways are “accessory” pathways and not essential for brain drainage there is no risk to the brain by a transvenous treatment.

## Conclusion

In summary, DAVFs of the ACV and PCV are rare. The venous anatomy of the skull base is a highly variable system and exact knowledge of the anatomy is essential to decide the best treatment option for these patients. We described three rare anatomical variants that might have clinical and therapeutic implications. Transvenous embolization is safe and effective and should be considered as the first-line treatment approach for condylar DAVFs.
